# 

**Published:** 1873-04

**Authors:** 


*Georgia Medical Association.*

*American Medical Association.*

The annual meeting will be held in St. Louis, on Wednesday, the 6th of May next.

THE Association of American Medical Editors will meet at St. Louis on the fifth of May.

*Communications.*

The following communications have been received: "Eclampsia Infantum," by T. Curtis Smith, M.D., of Ohio; "A Dichotomous Analysis of Diseases, Founded on the Symptoms," by W. T. Grant, M.D., of Georgia; "Medical Extracts: Practical and Philosophical," No. 2, by L. B. Anderson, M.D., of Virginia. Correspondence—"Uterine Hemorrhage," by John H. Weir, M.D., of Illinois; "Vegetable Fungi Growing in the Human Ear—Aspergillus Nigricans, or Mykomyringitis," by A. R. Kilpatrick, M.D., of Texas.

*List of Payments Received for the Record, Commencing with the January Number.*

Georgia.—Dr. E. B. Rees, 2 50; Dr. W. Barton, 1 50; Dr. A. B. Calhoun, 2 50; Dr. W. R. Burgess, 2 50; Dr. P. H. Wright, 2 50; Dr. J. G. McCrary, 2 50; Dr. C. B. Nottingham, 2 50; Dr. P. E. L. Jennings, 2 50; Dr. L. C. Wisdom, 2 50; Dr. A. J. Logan, 2 50; Dr. C. A. W. Bostic, fifty cents, balance; Dr. W. A. Culbertson, 2 50; Dr. W. F. Holt, 2 50; Dr. J. C. Sims, 2 50; Dr. John Hardeman, 2 00; Dr. C. M. Bold, 2 50; Dr. Jos. Underwood, 2 50.

Mississippi.—Dr. E. T. Easley, $2 50; Dr. N. B. Warren, 2 50; Dr. Z. J. Scott, 2 50; Dr. C. R. Smith, 2 50; Dr. P. M. Catchings, 2 50; Dr. J. S. Green, 2 50; Dr. J. R. Green, 2 50; Dr. J. A. Johnson, 2 50; Dr. E. D. Yeates, 2 50; Dr. John N. Rhodes, 2 50; Dr. R. Collins, 2 50; Dr. E. S. Lanier, 2 50; Dr. James T. Alford, 2 50; Dr. J. W. Ethridge, 2 50; Dr. William Giles, 2 50; Dr. S. Nic. Walker, 2 50.

Alabama.—Dr. J. D. Harrell, 2 50; Dr. M. D. Liles, 2 50; Dr. C. A. Henderson, 2 50; Dr. W. J. Caldwell, 2 50; Mr. Thos. Merriwether, 2 50; Dr. James McDade, 2 50; Dr. F. B. Henderson, 2 50; Dr. C. P. Sanders, 2 50; Dr. A. G. Grove, 2 50.

North Carolina.—Dr. G. T. Scarburg, 2 50; Dr. J. J. Bruton, 2 50; Dr. L. W. Coleman, 2 50; Dr. R. V. Cowan, 2 50; Dr. H. Kelly, fifty cents, balance; Dr. M. W. Hill, fifty cents, balance.

Louisiana.—Dr. J. W. Saunders, 2 50; Dr. P. W. Callihan, 2 50; Dr. John S. Davis, 2 50; Dr. S. L. Singletary, 2 00.

Arkansas.—Drs. Hodge and Hodge, 2 50; Dr. Edward Parham, 2 50.

Texas.—Dr. T. H. Taylor, 2 50; Dr. H. B. Lain, 2 50.

Virginia.—Dr. J. A. Alexander, fifty cents, balance.

South Carolina.—Dr. John S. Hughson, 2 50.

Illinois.—Dr. John W. Weir, 2 50.

Oregon.—Dr. W. A. Cusick, 2 50.

### The Southern Medical Record.

EDITED BY

T. S. POWELL, M.D.,

AND

W. T. GOLDSMITH, M.D.

ASSOCIATE EDITORS:

GEORGIA.

Robert Battey, M.D.
R. C. Word, M.D.
W. L. Kirkpatrick, M.D.
James A. Long, M.D.
W. L. M. Harris, M.D.
H. L Battle, M.D.
W. C. Musgrove, M D
L B. Bouchelle, M.D.
John C. Drake, M.D.
E. F. Knott, M.D.
Thomas Burdell, M.D.
E. H. W. Hunter, M.D.
V. S. Hitt, M.D.
J. E. Pope, M.D.
J. A. Hunnicutt, M.D.
A. H. Brantley, M.D.
J. J. Knott, M.D.
J. C. Wisdom, M.D.
W. W. Leake, M.D.

ALABAMA.

J. C. Knox, M.D.
Geo. F. Taylor, M.D.
J. B. Barnett, M.D.
S. M. Hogan, M.D.
D. S. Hopping, M.D.
J. T. Searcy, M.D.
S. F. Hill, M.D.

MISSISSIPPI.

C. B. Gallaway, M.D.
Edward Lea, M.D.
S. V. D. Hill, M.D.
W. B. Harvey, M.D.
J. W. M. Shattuck, M.D.
E. T. Henry, M.D.
T. P. Lockwood, M.D.
Robert Kells, M.D.

VIRGINIA.

Charles S. Lewis, M.D.
John H. Claiborne, M.D.
F. Horner, Jr., M.D.
R. J. Preston, M.D.
A. S. Payne, M.D.
J. E. Lockridge, M.D.
J. S. Wharton, M.D.
Wm. O. Owen, M.D.
Sam'l P. Ripley, M.D.
Benj. Blackford, M.D.
E. A. Drewry, M.D.
Rob B. Tunstall, M.D.
A. J. Terrell, M.D.
W. F. Barr, M.D.
L. B. Anderson, M D.
S. L. Jepson, M.D., W Va.
J. M. Lazzell, M.D.

SOUTH CAROLINA.

E. T. McSwain, M.D.
G. M. Rivers, M.D.

TENNESSEE.

J. D. Tucker, M.D.
Swan M. Burnett, M.D.

TEXAS.

S. M. Welch, M.D
T. J. Heard, M.D.
W. A. Heard, M.D.

ARKANSAS.

A. D. Hodge, M.D.
J. K. Hodge, M.D.

NORTH CAROLINA.

J. R. Hughes, M.D.
S. S. Satchwell, M.D.
A. B. Pierce, M.D.
H. Kelly, M.D.
Geo. A. Foote, M.D.
E. A. Anderson, M.D.
H. T. Bahnson, M.D.
Willis Alston, M.D.
Thos. F. Wood, M.D.
N. J. Pittman, M.D.

CORRESPONDING EDITORS:

J. M. Toner, M.D., Washington City, D. C.
John H. Weir, M.D., Illinois.
Thomas J Gallaher, M. D, Pennsylvania.
Ely Van DeWarker, M. D., New York.
Robert Robson, M D., Indiana.
C. C. F. Gay, M.D , New York.
*Surgeon of Buffalo General Hospital.*
W. T. Taylor, M.D , Kentucky.
J. Orne Green, Boston, Massachusets.
B. W. Stone, M.D., Kentucky.
*Assistant Physician Western Lunatic Asylum.*
James Tyson, M.D., Philadelphia, Pa.
W. W. Leen, M.D., Philadelphia.
M. H. Henry, M.D., New York,
*Surgeon to New York Dispensary, Department of Venerial and Skin Diseases.*
Wm. R. Whitehead, M.D., Colorado Territory.
A. Patton, M.D., Indiana.
Benjamin Howard, M.D., New York.
Chas Kearns, M.D., Kentucky.
S. C. Busey, M.D., Washington, D. C.
A. W. Hobson, M.D., Arkansas.
A. W. Calhoun, M.D., now of Berlin, Prussia.
T. Curtis Smith, Ohio.
Geo. Strawbridge, M.D., Philadelphia.

---

☞ *All Business Letters should be addressed to POWELL & GOLDSMITH, Post Office Box 527, Atlanta, Georgia.*

